# Dyslipidemia Risk in Thyroid Cancer Patients: A Nationwide Population-Based Cohort Study

**DOI:** 10.3389/fendo.2022.893461

**Published:** 2022-06-27

**Authors:** Youhyun Song, Hye Sun Lee, Goeun Park, Sang-Wook Kang, Ji Won Lee

**Affiliations:** ^1^ Department of Family Medicine, Gangnam Severance Hospital, Yonsei University College of Medicine, Seoul, South Korea; ^2^ Biostatistics Collaboration Unit, Yonsei University College of Medicine, Seoul, South Korea; ^3^ Thyroid-Endocrine Surgery Division, Department of Surgery, Yonsei University College of Medicine, Seoul, South Korea

**Keywords:** thyroid, thyroid cancer, dyslipidemia, hypercholesterolemia, lipids

## Abstract

**Objective:**

Thyroid cancer (TC) prevalence has been rapidly increasing. While the relationship between thyroid hormones and lipids has been widely investigated, studies regarding dyslipidemia in patients with TC have been scarce and controversial. We aimed to investigate dyslipidemia risk after TC diagnosis compared to the general population without TC.

**Method:**

A population-based prospective study was conducted using data from the Korean National Health Insurance Service-National Sample Cohort Database 2.0 (NHIS-NSC DB 2.0), with health insurance claim data of 1,108,369 subjects between 2002 and 2015. The final study sample comprised 466,735 adult subjects without TC or dyslipidemia diagnoses before the index year, 2009. Bidirectional analyses were performed using prospective and retrospective concepts. In the prospective analysis, Kaplan-Meier estimates were calculated and log-rank tests and univariable and multivariable Cox regression analyses were performed to determine the relationship between TC and dyslipidemia. The retrospective analysis involved 1:5 nested case-control matching based on dyslipidemia status and conditional logistic regression analysis.

**Results:**

No significant difference in dyslipidemia incidence was observed between TC patients and the control group, in either the prospective matched (log-rank P = 0.483) or non-matched (log-rank P = 0.424) analyses, or the retrospective analysis (P = 0.3724). In the prospective analysis, 193 patients after TC diagnosis showed similar risk of developing dyslipidemia with the 466,542 controls during the median 7 years of follow-up (unadjusted hazard ratio [HR], 1.102; 95% confidence interval [CI], 0.878-1.383; adjusted HR, 0.932; 95% CI, 0.707-1.230). Multiple propensity score-adjusted models showed similar results, and 114 patients and 570 matched controls showed an HR of 0.818 (95% CI, 0.598-1.120). In the retrospective comparison of dyslipidemia risk in 170 patients and 277,864 controls, the odds ratio was 0.822 (95% CI, 0.534-1.266).

**Conclusions:**

Dyslipidemia risk was not significantly different between patients with TC and the general population, in both prospective and retrospective analyses.

## Introduction

Thyroid cancer (TC) is the most common endocrine malignancy, and its incidence has been rapidly increasing both globally and in Korea (1). However, despite this increase in incidence, owing to the high survival rates and early detection, the overall life expectancy of patients is relatively longer than that of patients with most other types of cancers (2–4). Therefore, long-term health management is essential for those who have completed initial TC treatment. The incidence of cardiometabolic diseases has also been increasing steadily worldwide. Recent analyses have shown that cardiovascular disease (CVD) is a leading cause of death among patients with TC, except for TC-related or other malignancy-related mortality (5, 6). Therefore, prevention, screening, and appropriate management of CVD risk factors are imperative in patients with TC.

Dyslipidemia has become the leading modifiable risk factor for CVD and has detrimental effects on certain types of cancer risk as well as prognosis (7–11). Thyroid hormones play a major role in lipid metabolism, and many studies have found relationships between thyroid diseases and lipids. Hyperthyroidism or thyrotoxicosis is associated with a depletion in the lipid stores and a decrease in blood lipid levels. Moreover, opposite changes occur in conditions resulting from thyroid hormone deficiency (12–14). However, only few studies have investigated dyslipidemia risk in patients with TC, and these have yielded conflicting results (15–24), possibly because of differences in the study population, sample size, and follow-up period.

Therefore, this study aimed to identify new-onset dyslipidemia risk in patients after TC diagnosis compared to the general population using a large cohort database representative of the Korean population. Furthermore, we performed prospective and retrospective analyses simultaneously to confirm the relationship between TC and dyslipidemia risk.

## Materials and Methods

### Study Design and Population

In this retrospective study of a prospective cohort, we used data from 1,108,369 subjects with health insurance claims in the Korean National Health Insurance Service-National Sample Cohort (NHIS-NSC) Database (DB) 2.0, between January 1, 2002 and December 31, 2015. Briefly, the NHIS is a universal and mandatory health insurance program for all Korean nationals. The NHIS-NSC is a population-based cohort comprising around one million individuals (approximately 2.2% of the general Korean population) retaining insurance entitlement, and is of appropriate sample size. Selection was done *via* stratified systematic random sampling based on age, sex, income level, and eligibility status to represent the entire Korean population. The NHIS-NSC DB is comprised of multiple datasets including sociodemographic information, health insurance claims (with the International Classification of Diseases, Tenth Revision [ICD-10] codes, admission and discharge, and treatment such as surgery or medication prescriptions), and the Korean National Health Screening Program. Detailed information on the NHIS-NSC is provided elsewhere (25).

**Figure 1:**
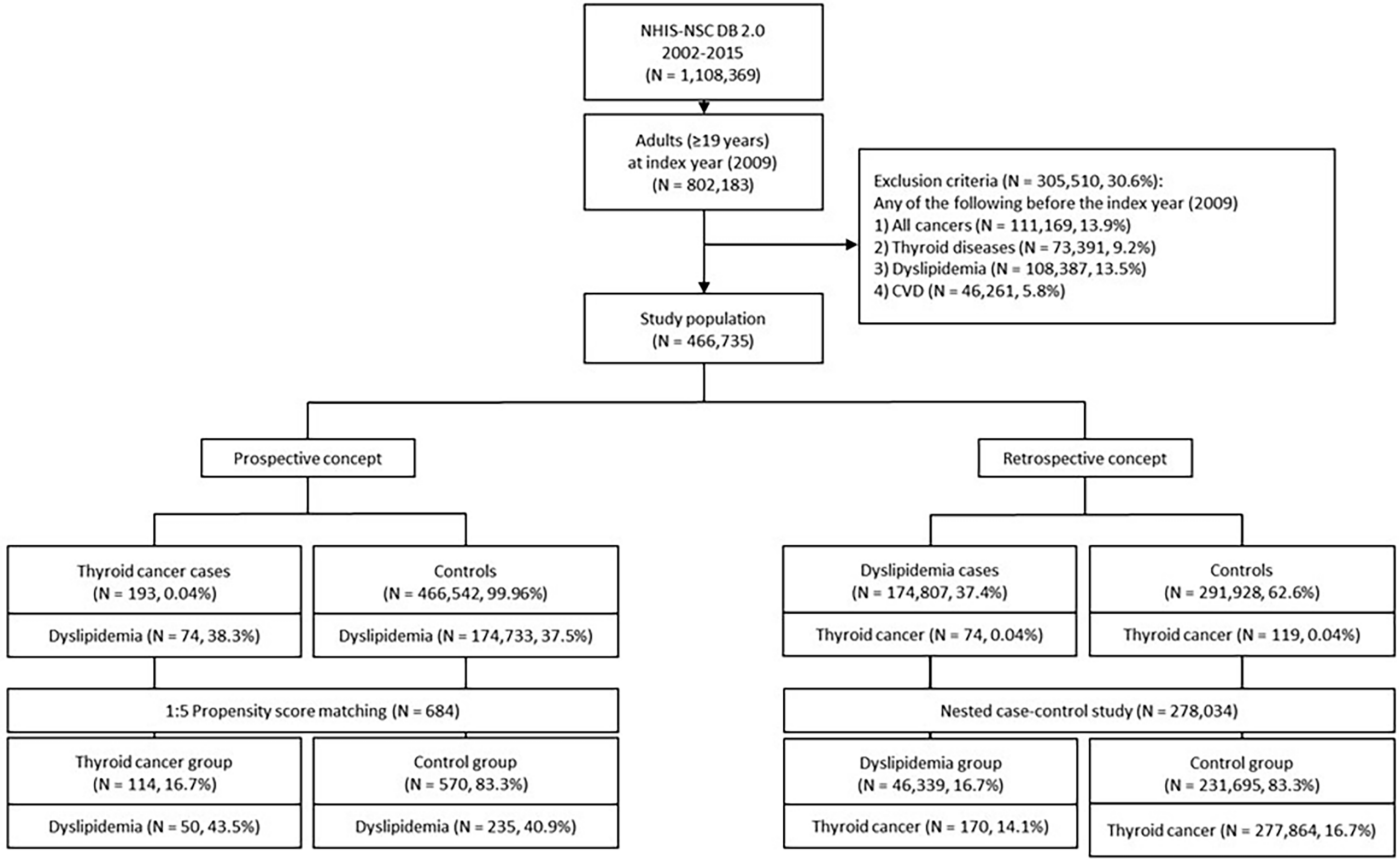
Flowchart illustrating the study design. NHIS-NSC DB, National Health Insurance Service-National Sample Cohort Database; CVD, cardiovascular disease.

The index year was set at 2009, and 802,183 adults (subjects aged 19 years and older in 2009) were included from the total 1,108,369 subjects in the database. The wash-out period was 2002 to 2008: we excluded 305,510 patients with history of any type of cancer including TC, dyslipidemia, thyroid-related disease, or CVD before the index year 2009 (see *Study Definitions and Outcomes *for diagnostic criteria). Within the final study population (n = 466,735) we performed bidirectional analysis using prospective and retrospective concepts to assess new-onset dyslipidemia in thyroid cancer patients, as well as whether if dyslipidemia is marker of underlying thyroid cancer. In the prospective analysis, the patients with TC (cases) and those without TC (controls) were divided on the basis of their diagnosis of TC in the index year 2009. An initial non-matched analysis was performed, and subsequent secondary analyses were performed after 1:5 propensity score matching. In the retrospective analysis, subjects were first divided on the basis of their dyslipidemia status (diagnosis between 2009 and 2015), and then a 1:5 nested case-control study was conducted. Subjects were followed up from baseline to the first dyslipidemia diagnosis, or December 31, 2015 (see **Figure 1** for a flowchart depicting the overall study design).

This study was performed in accordance with the tenets of the Declaration of Helsinki and was approved by the Institutional Review Board of Yonsei University Gangnam Severance Hospital (approval number: 3-2019-0370). The requirement for informed consent was waived because this retrospective observational study was based on an anonymous de-identified dataset provided by the NHIS.

### Study Definitions and Outcomes

Hypertension was defined as ICD-10 codes I10-I15, the prescription of an antihypertensive drug, systolic blood pressure of ≥140 mmHg or diastolic blood pressure of ≥90 mmHg on a health examination, current use of an antihypertensive drug, or a diagnosis by a physician. Diabetes mellitus was defined as ICD-10 codes E10-E14, the prescription of an antidiabetic drug, fasting blood glucose of ≥126 mg/dl, hemoglobin A1c of ≥6.5% (48 mmol/mol), current use of an antidiabetic drug or insulin, or a diagnosis by a physician.

Dyslipidemia was defined as ICD-10 code E78, the prescription of lipid-lowering drugs (statins, fibrates, or combinations) for ≥20 days a month or for ≥3 months, a diagnosis by a physician, current use of lipid-lowering medications, or a diagnosis according to the National Cholesterol Education Program-Adult Treatment Panel III criteria: (1) hypercholesterolemia (serum total cholesterol ≥ 240 mg/dl), (2) hypertriglyceridemia (serum triglyceride ≥ 200 mg/dl), (3) hyper-low-density-lipoprotein [LDL] cholesterolemia (serum LDL-C ≥ 160 mg/dl), and (4) hypo-high-density-lipoprotein [HDL] cholesterolemia (serum HDL-C < 40 mg/dl).

TC was defined as ICD-10 code C73 (malignant neoplasm of the thyroid gland), and cancer was defined as ICD-10 codes C00-C97 (excluding C73). Thyroid-related diseases were defined as ICD-10 codes E03-E07.

CVD was defined as acute myocardial infarction (I21), stroke (I60-I63), heart failure (I50), or angina (I20). Body mass index (BMI) was defined as weight(kg)/height^2^(m^2^). Lifestyle factors were defined as follows: smoking (current or non/ex-smoker), drinking (non-drinker or drinker), and exercise (none or any days a week). Economic status was evaluated based on health insurance premium level.

### Statistical Analysis

In this study, we performed statistical analyses on the basis of two concepts: prospective and retrospective. In the prospective analysis, we divided the baseline study population into the case and control groups (as described previously) and compared the incidence of new-onset dyslipidemia between the two groups. In the retrospective analysis, we divided the study population into patients diagnosed with dyslipidemia between 2009-2015 and subjects without dyslipidemia, and then investigated the presence or absence of TC among these subjects.

In the prospective analysis, Kaplan-Meier estimates were calculated and log-rank tests were performed. Univariable and multivariable Cox regression analyses were also performed to determine the relationship between TC and dyslipidemia. To reduce the potential effects of confounding factors and baseline selection bias, propensity scores were computed for each subject with multivariable logistic regression including age, sex, body mass index (BMI), hypertension, diabetes mellitus, and lifestyle factors (smoking, drinking, and exercise) in 2009. Thereafter, we divided the data into three strata defined by tertiles of propensity scores. Stratified Cox regression was employed and Cox regression was performed within each stratum. Inverse probability treatment weighting (IPTW) was considered, and 1:5 propensity score matching was conducted between the patients with TC and subjects without TC by employing clustered Cox regressions.

In the retrospective analysis, 1:5 nested case-control matching was conducted between patients diagnosed with dyslipidemia and subjects without dyslipidemia. As matching variables, age, sex, BMI, hypertension, diabetes mellitus, lifestyle factors (smoking, drinking, and exercise), and in addition follow-up periods (varied according to period of dyslipidemia diagnosis) were considered. Conditional logistic regression was performed to confirm the relationship between dyslipidemia and TC.

All data are presented as mean ± standard deviation or number (percentage, %). To compare the baseline covariates between the two groups, Student’s t-test for continuous variables and chi-square test for categorical variables were performed. Two-sided P values of <0.05 were considered statistically significant. All analyses were performed using SAS Enterprise Guide 7.13 (SAS Incorporated, Cary, NC, USA) and R package version 4.0.2 (http://www.R-project.org).

## Results

Of the 1,108,369 initial subjects, 466,735 were included in the final study sample after applying the inclusion and exclusion criteria. The median follow-up period was 7 years for all analyses. In prospective analysis, the case group, i.e., patients diagnosed with TC in 2009, included 193 patients (0.04%), and the control group included the remaining subjects (n = 466,542, 99.96%). After 1:5 propensity score matching between the two groups, 684 subjects (114 [16.7%] cases and 570 [83.3%] controls) were analyzed. In the retrospective analysis, the study sample was first divided on the basis of their dyslipidemia status, with 174,807 (37.4%) having dyslipidemia and 291,928 (62.6%) without dyslipidemia. After 1:5 nested case-control matching, the data of 278,034 subjects (46,339 [16.7%] with dyslipidemia and 231,695 [83.3%] without) were analyzed.

### Baseline Characteristics of the General Study Population

The mean age of the final study sample of 466,735 subjects was 39.97 ± 14.44 years, and 255,630 (54.77%) were men. Compared with subjects without TC (n = 466,542), patients with TC (n = 193) were more likely to be older, female, non-smokers, non-drinkers, and have hypertension and lower triglyceride and LDL values. No significant differences in mean BMI, fasting glucose level, or diabetes incidence were observed between the two groups. Among the 193 patients with TC, 159 (82.39%) had undergone total thyroidectomy or hemithyroidectomy, 109 (56.48%) had undergone radioactive iodine treatment, and 174 (90.16%) were on levothyroxine replacement. Compared with subjects without dyslipidemia (n = 291,928), patients with dyslipidemia (n = 174,807) were more likely to be older, male, smokers or drinkers, and have higher mean BMI and incidence of hypertension or diabetes mellitus (**Table 1**).

**Table 1:** Baseline characteristics of the general study population according to the thyroid cancer and dyslipidemia statuses.

Variable	Baseline study sample								
	Total (N = 466,735)	Thyroid cancer status		Standardized difference	P-value	Dyslipidemia status		Standardized difference	P-value
		Case (N = 193)	Control (N = 466,542)			Case b (N = 174,807)	Control (N = 291,928)		
Age (n = 466,735)	39.97 ± 14.44	43.09 ± 11.11	39.96 ± 14.44	-0.24	**0.0001**	44.36 ± 13.75	37.33 ± 14.19	-0.50	**<.0001**
Sex (n = 466,735)					**<.0001**				**<.0001**
Male	255,630 (54.77)	37 (19.17)	255,593 (54.78)	0.79		111,658 (63.88)	143,972 (49.32)	-0.30	
Female	211,105 (45.23)	156 (80.83)	210,949 (45.22)	-0.79		63,149 (36.12)	147,956 (50.68)	0.30	
BMI (n = 227,202)	23.23 ± 3.23	23.08 ± 3.08	23.23 ± 3.23	0.05	0.625	24.08 ± 3.15	22.39 ± 3.09	-0.54	**<.0001**
SBP (n = 113,150)	121.18 ± 14.55	119.49 ± 14.75	121.18 ± 14.55	0.12	0.389	123.49 ± 14.69	118.14 ± 13.78	-0.38	**<.0001**
DBP (n = 113,151)	75.79 ± 9.91	74.38 ± 9.38	75.79 ± 9.91	0.15	0.2928	77.29 ± 9.94	73.81 ± 9.50	-0.36	**<.0001**
FBS (n = 113,156)	94.93 ± 21.31	93.31 ± 14.28	94.93 ± 21.32	0.09	0.4034	97.49 ± 24.62	91.56 ± 15.32	-0.29	**<.0001**
TCh (n = 113,156)	188.65 ± 40.20	185.04 ± 20.59	188.65 ± 40.21	0.11	0.199	196.96 ± 46.93	177.71 ± 25.20	-0.51	**<.0001**
TG (n = 113,153)	125.67 ± 89.50	102.51 ± 38.96	125.69 ± 89.52	0.34	**<.0001**	156.06 ± 104.72	85.71 ± 36.21	-0.90	**<.0001**
nHDL (n = 113,139)	131.84 ± 49.62	130.53 ± 20.78	131.84 ± 49.63	0.03	0.6422	143.00 ± 58.29	117.16 ± 29.17	-0.56	**<.0001**
HDL (n = 113,139)	56.81 ± 31.50	54.51 ± 10.28	56.81 ± 31.50	0.10	0.103	53.97 ± 38.14	60.56 ± 18.93	0.22	**<.0001**
LDL (n = 113,109)	120.03 ± 270.88	109.95 ± 21.41	120.04 ± 270.95	0.05	**0.0013**	135.11 ± 358.10	100.21 ± 23.74	-0.14	**<.0001**
Hypertension (n = 466,735)					**0.0091**				**<.0001**
No	431,765 (92.51)	169 (87.56)	431,596 (92.51)	0.17		151,292 (86.55)	280,473 (96.08)	0.34	
Yes	34,970 (7.49)	24 (12.44)	34,946 (7.49)	-0.17		23,515 (13.45)	11,455 (3.92)	-0.34	
Diabetes mellitus (n = 466,735)					0.5776				**<.0001**
No	454,016 (97.27)	189 (97.93)	453,827 (97.27)	-0.04		165,554 (94.71)	288,462 (98.81)	0.23	
Yes	12,719 (2.73)	4 (2.07)	12,715 (2.73)	0.04		9253 (5.29)	3466 (1.19)	-0.23	
Smoking status (n = 222,405)					**<.0001**				**<.0001**
No	154,463 (69.45)	108 (90.76)	154,355 (69.44)	-0.55		72,207 (64.99)	82,256 (73.90)	0.19	
Yes	67,942 (30.55)	11 (9.24)	67,931 (30.56)	0.55		38,893 (35.01)	29,049 (26.10)	-0.19	
Drinking status (n = 215,357)					**<.0001**				**<.0001**
No	96,882 (44.99)	76 (66.09)	96,806 (44.98)	-0.43		47,495 (43.90)	49,387 (46.09)	0.04	
Yes	118,475 (55.01)	39 (33.91)	118,436 (55.02)	0.43		60,702 (56.10)	57,773 (53.91)	-0.04	
Exercise status (n = 214,850)					0.8806				**<.0001**
No	83,337 (38.79)	45 (39.47)	83,292 (38.79)	-0.01		38,540 (35.70)	44,797 (41.91)	0.13	
Yes	131,513 (61.21)	69 (60.53)	131,444 (61.21)	0.01		69,428 (64.30)	62,085 (58.09)	-0.13	
Insurance premium (n = 458,111)					**0.0001**				**<.0001**
Low	114,567 (25.01)	31 (16.49)	114,536 (25.01)	0.21		40,935 (23.87)	73,632 (25.69)	0.04	
Middle	186,658 (40.75)	66 (35.11)	186,592 (40.75)	0.12		70,704 (41.24)	115,954 (40.45)	-0.02	
High	156,886 (34.25)	91 (48.40)	156,795 (34.24)	-0.29		59,824 (34.89)	97,062 (33.86)	-0.02	
Type of thyroidectomy (n = 193)									
Thyroidectomy		136 (70.47)				57 (77.03)	79 (66.39)		
Hemithyroidectomy		23 (11.92)				9 (12.16)	14 (11.76)		
None		34 (17.62)				8 (10.81)	26 (21.85)		
Levothyroxine dosage (n = 174)									
Tertile 1 (<1.000)		45 (25.86)				14 (20.29)	31 (29.52)		
Tertile 2 (<1.279)		71 (40.80)				31 (44.93)	40 (38.10)		
Tertile 3 (≥1.279)		58 (33.33)				24 (34.78)	34 (32.38)		
RAI treatment (n = 193)									
No		84 (43.52)				31 (41.89)	53 (44.54)		
Yes		109 (56.48)				43 (58.11)	66 (55.46)		

Results are shown as mean ± standard deviation or frequency (%).

Matched for age, sex, BMI, hypertension, diabetes mellitus, smoking, drinking, exercise, and insurance premium. Values in bold are significant.

BMI, body mass index; SBP, systolic blood pressure; DBP, diastolic blood pressure; FBS, fasting blood sugar; TCh, total cholesterol; TG, triglyceride; nHDL, nonhigh-density lipoprotein; HDL, high-density lipoprotein; LDL, low-density lipoprotein; RAI, radioactive iodine.

### Prospective and Retrospective Analysis Groups

In the prospective analysis, after 1:5 propensity score matching, the mean age of the total 684 matched cases (n = 114) and controls (n = 570) was 45.07 ± 12.97 years, and 152 (22.22%) were men. The retrospective analysis included a total of 278,034 subjects with (n = 46,339) and without dyslipidemia (n = 231,695) after 1:5 nested case-control matching; their mean age was 40.42 ± 9.29 years, and 175,458 (63.11%) were men. All characteristics between the two groups were matched and well-balanced, with standardized differences below ±0.1 (**Table 2**).

**Table 2:** Characteristics of the prospective and retrospective analysis groups.

Variable	Prospective concept (1:5 propensity score matching)					Retrospective concept (nested case-control 1:5 matching)				
	Total (N = 684)	Thyroid cancer status		Standardized difference	P-value	Total (N = 278,034)	Dyslipidemia status		Standardized difference	P-value
		Case (N = 114)	Controls (N = 570)				Case (N = 46,339)	Controls (N = 231,695)		
Age (n = 684 and 278,034)	45.07 ± 12.97	45.11 ± 10.51	45.06 ± 13.41	0.00	0.9593	40.42 ± 9.29	40.42 ± 9.29	40.42 ± 9.29	0.00	>.9999
Sex					0.8693					>.9999
Male	152 (22.22)	26 (22.81)	126 (22.11)	-0.02		175,458 (63.11)	29,243 (63.11)	146,215 (63.11)	0.00	
Female	532 (77.78)	88 (77.19)	444 (77.89)	0.02		102,576 (36.89)	17,096 (36.89)	85,480 (36.89)	0.00	
BMI (n = 684 and 278,034)	23.05 ± 3.16	23.09 ± 3.07	23.04 ± 3.19	-0.02	0.8735	23.25 ± 2.25	23.25 ± 2.25	23.25 ± 2.25	0.00	0.88
SBP (n = 368 and 140,932)	120.68 ± 15.92	119.49 ± 14.75	120.89 ± 16.13	0.09	0.5481	118.13 ± 11.12	118.79 ± 11.13	117.97 ± 11.11	-0.07	**<.0001**
DBP (n = 368 and 140,935)	75.28 ± 10.17	74.38 ± 9.38	75.44 ± 10.31	0.11	0.4786	74.20 ± 8.29	74.80 ± 8.28	74.05 ± 8.28	-0.09	**<.0001**
FBS (n = 368 and 140,938)	92.22 ± 13.31	93.31 ± 14.28	92.03 ± 13.15	-0.09	0.5103	91.33 ± 10.97	91.90 ± 11.26	91.18 ± 10.89	-0.06	**<.0001**
TCh (n = 368 and 140,932)	187.65 ± 29.70	185.04 ± 20.59	188.11 ± 31.03	0.12	0.3522	186.27 ± 30.95	195.56 ± 39.98	183.94 ± 27.76	-0.34	**<.0001**
TG (n = 368 and 140,930)	112.60 ± 74.36	102.51 ± 38.96	114.37 ± 78.87	0.19	0.0873	113.21 ± 71.35	151.88 ± 100.88	103.53 ± 57.87	-0.59	**<.0001**
nHDL (n = 368 and 140,912)	129.53 ± 36.09	130.53 ± 20.78	129.36 ± 38.17	-0.04	0.7414	57.12 ± 26.34	54.16 ± 38.37	57.86 ± 22.27	0.12	**<.0001**
HDL (n = 368 and 140,912)	58.11 ± 24.87	54.51 ± 10.28	58.75 ± 26.58	0.21	**0.0394**	57.12 ± 26.34	54.16 ± 38.37	57.86 ± 22.27	0.12	**<.0001**
LDL (n = 368 and 140,899)	114.02 ± 95.29	109.95 ± 21.41	114.73 ± 102.94	0.06	0.4617	113.12 ± 186.05	138.42 ± 396.76	106.79 ± 60.51	-0.11	**<.0001**
Hypertension					>.9999					>.9999
No	570 (83.33)	95 (83.33)	475 (83.33)	0.00		277,014 (99.63)	46,169 (99.63)	230,845 (99.63)	0.00	
Yes	114 (16.67)	19 (16.67)	95 (16.67)	0.00		1020 (0.37)	170 (0.37)	850 (0.37)	0.00	
Diabetes mellitus					0.7385					>.9999
No	668 (97.66)	111 (97.37)	557 (97.72)	0.02		278,028 (100.00)	46,338 (100.00)	231,690 (100.00)	0.00	
Yes	16 (2.34)	3 (2.63)	13 (2.28)	-0.02		6 (0.00)	1 (0.00)	5 (0.00)	0.00	
Smoking status					>.9999					>.9999
No	618 (90.35)	103 (90.35)	515 (90.35)	0.00		173,856 (63.67)	28,976 (63.67)	144,880 (63.67)	0.00	
Yes	66 (9.65)	11 (9.65)	55 (9.65)	0.00		99,216 (36.33)	16,536 (36.33)	82,680 (36.33)	0.00	
Drinking status					0.6083					>.9999
No	464 (67.84)	75 (65.79)	389 (68.25)	0.05		98,940 (36.29)	16,490 (36.29)	82,450 (36.29)	0.00	
Yes	220 (32.16)	39 (34.21)	181 (31.75)	-0.05		173,688 (63.71)	28,948 (63.71)	144,740 (63.71)	0.00	
Exercise status					0.8884					>.9999
No	266 (38.89)	45 (39.47)	221 (38.77)	-0.01		73,764 (27.06)	12,294 (27.06)	61,470 (27.06)	0.00	
Yes	418 (61.11)	69 (60.53)	349 (61.23)	0.01		198,864 (72.94)	33,144 (72.94)	165,720 (72.94)	0.00	
Insurance premium					0.8207					>.9999
Low	107 (15.64)	18 (15.79)	89 (15.61)	0.00		40,878 (14.70)	6813 (14.70)	34,065 (14.70)	0.00	
Middle	239 (34.94)	37 (32.46)	202 (35.44)	0.06		130,266 (46.86)	21,711 (46.86)	108,555 (46.86)	0.00	
High	338 (49.42)	59 (51.75)	279 (48.95)	-0.06		106,860 (38.44)	17,810 (38.44)	89,050 (38.44)	0.00	
Type of thyroidectomy										
Thyroidectomy		80 (70.18)					260 (0.56)			
Hemithyroidectomy		16 (14.04)					86 (0.19)			
None		18 (15.79)					45,993 (99.25)			
Levothyroxine dosage										
Tertile 1 (<1.000)		26 (25.74)					2 (9.09)			
Tertile 2 (<1.279)		41 (40.59)					11 (50.00)			
Tertile 3 (≥1.279)		34 (33.66)					9 (40.91)			
RAI treatment										
No		45 (39.47)					46,185 (99.67)			
Yes		69 (60.53)					154 (0.33)			

Results are shown as mean ± standard deviation or frequency (%).

Matched for age, sex, BMI, hypertension, diabetes mellitus, smoking, drinking, exercise, and insurance premium. Values in bold are significant.

BMI, body mass index; SBP, systolic blood pressure; DBP, diastolic blood pressure; FBS, fasting blood sugar; TCh, total cholesterol; TG, triglyceride; nHDL, nonhigh-density lipoprotein; HDL, high-density lipoprotein; LDL, low-density lipoprotein; RAI, radioactive iodine.

### Dyslipidemia Incidence in Patients With TC and Controls

**Figure 2:**
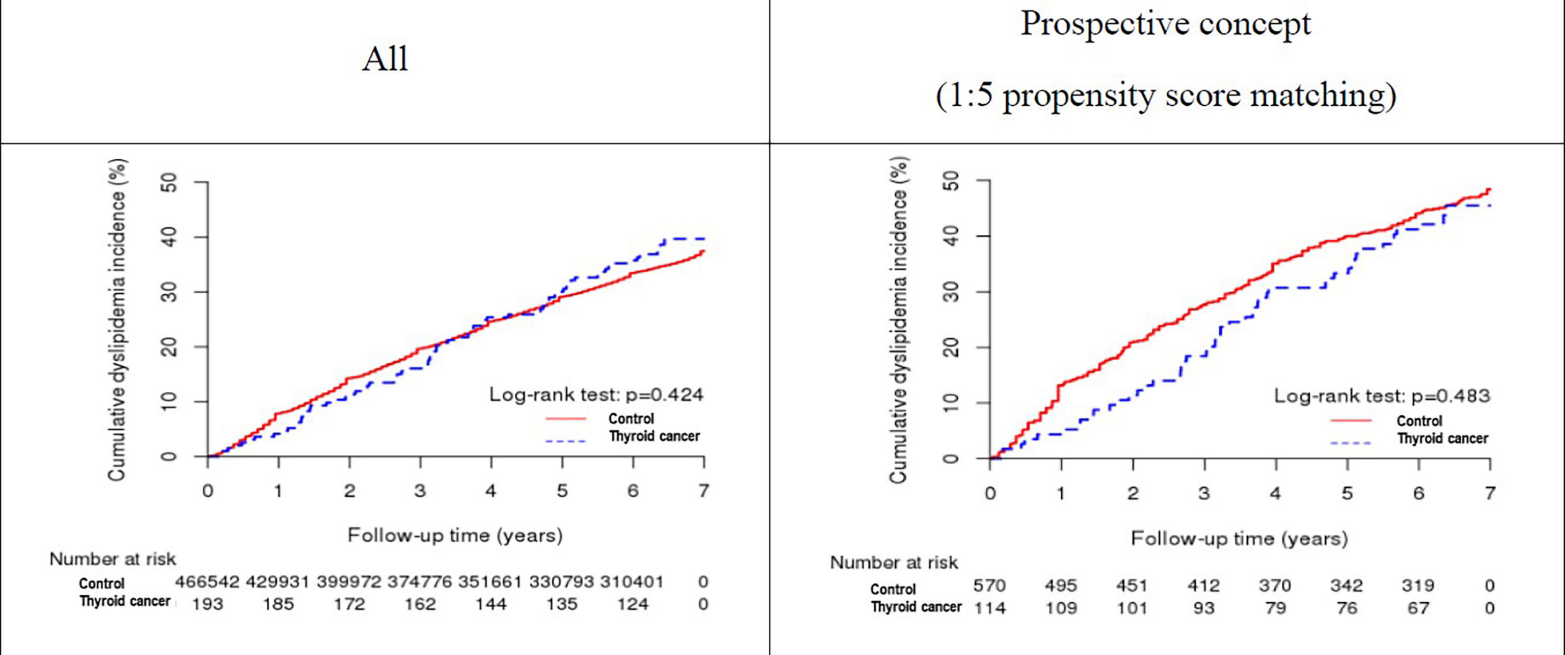
Kaplan-Meier curves showing dyslipidemia risk in patients with thyroid cancer.

**Figure 3:**
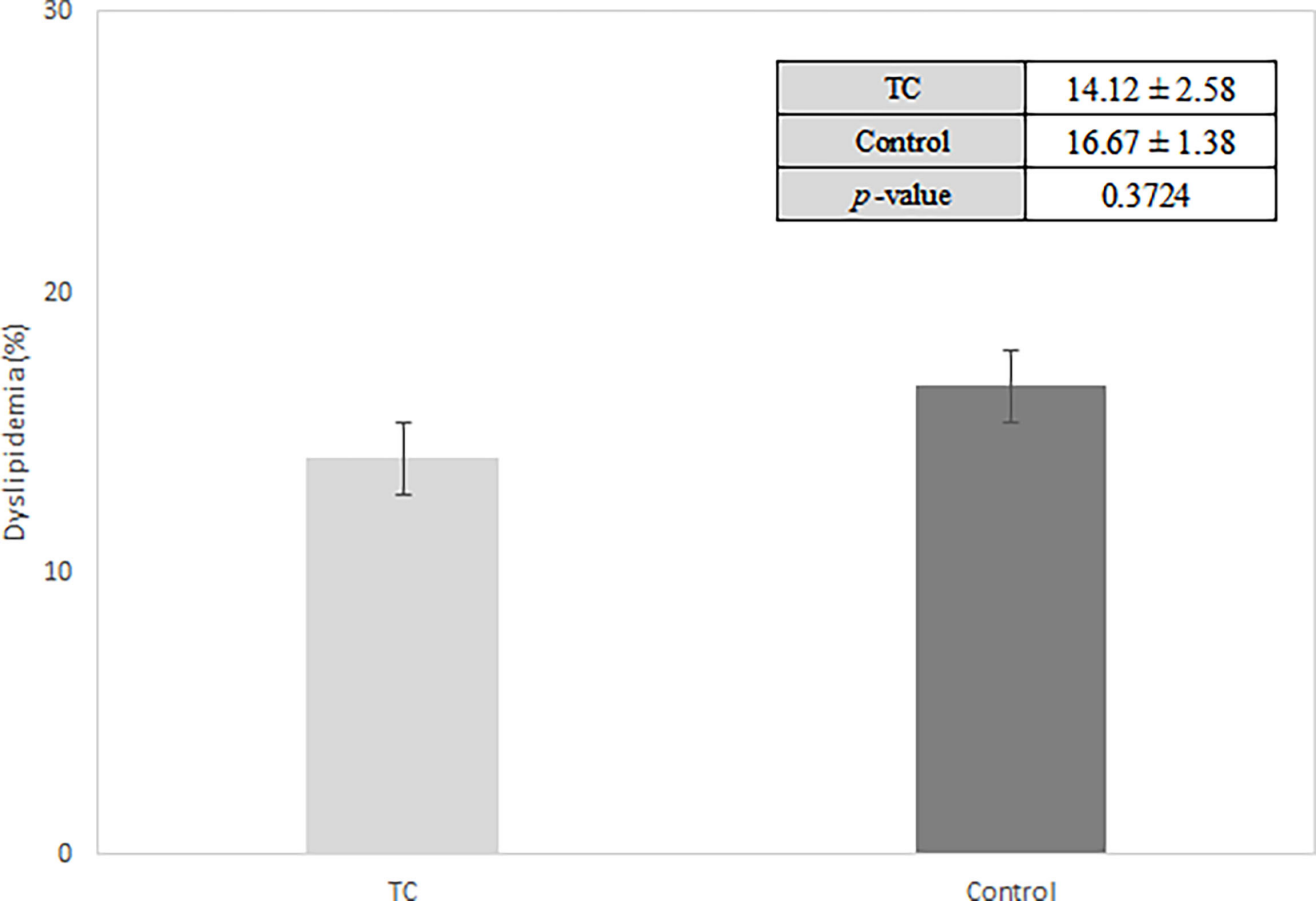
Bar plot showing dyslipidemia risk in patients with thyroid cancer. TC, Thyroid cancer; Values are presented as mean ± standard error.

During the median follow-up period of 7 years, the prospective unadjusted, matched, and retrospective analyses revealed that 74 (38.34%), 50 (43.86%), and 24 (14.12%) patients with TC, respectively, developed dyslipidemia, compared with 174,733 (37.45%), 276 (48.42%), and 46,315 (16.67%) subjects in the control group, respectively. The difference in dyslipidemia frequency between the case and control groups was not statistically significant in either of the three analyses (P = 0.7986, 0.3734, and 0.372, respectively) (**Table 3**). The Kaplan-Meier curves (**Figure 2**) and a bar plot (**Figure 3**) depict dyslipidemia incidence between the cases and controls. No significant difference in dyslipidemia incidence was observed between the two groups, in either the prospective matched (log-rank P = 0.483) or non-matched (log-rank P = 0.424) analyses, or the retrospective analysis (P = 0.3724).

**Table 3:** Comparison of dyslipidemia incidence between the patients with thyroid cancer and controls.

Outcome	All				Prospective concept (1:5 propensity score matching)				Retrospective concept (nested case-control 1:5 matching)			
	Total (N = 466,735)	TC (N = 193)	Control (N = 466,542)	P-value	Total (N = 690)	TC (N = 114)	Control (N = 575)	P-value	Total (N = 278,034)	TC (N = 170)	Control (N = 277,864)	P-value
	Frequency (%)	Frequency (%)	Frequency (%)		Frequency (%)	Frequency (%)	Frequency (%)		Frequency (%)	Frequency (%)	Frequency (%)	
Dyslipidemia				0.7986				0.3734				0.3724
No	291,928 (62.55)	119 (61.66)	291,809 (62.55)		358 (52.34)	64 (56.14)	294 (51.58)		231,695 (83.33)	146 (85.88)	231,549 (83.33)	
Yes	174,807 (37.45)	74 (38.34)	174,733 (37.45)		326 (47.66)	50 (43.86)	276 (48.42)		46,339 (16.67)	24 (14.12)	46,315 (16.67)	

### Risk of Dyslipidemia in Patients With TC and Controls

Dyslipidemia risk was estimated using various models using hazard ratios (HRs) (prospective analysis) or odds ratios (ORs) (retrospective analysis), in both the original and matched populations (**Table 4**). Overall, no significant difference in dyslipidemia risk was observed between the case and control groups irrespective of the analysis model. In the final study population, the patients with TC showed a similar risk to that of the controls with HRs of 1.102 (95% confidence interval [CI], 0.878-1.383; unadjusted) and 0.932 (95% CI, 0.707-1.230) when adjusted for multiple confounding variables. Similar trends in dyslipidemia development were demonstrated in the propensity score-adjusted models, with HRs of 0.964 (95% CI, 0.731-1.273) with stratification, 0.938 (95% CI, 0.711-1.238) with regression adjustment, 1.012 (95% CI, 0.838-1.223) with stabilized IPTW weighting, 0.818 (95% CI, 0.598-1.120) when matched, and within all the propensity score tertiles. Moreover, in the retrospective concept analysis, the OR of dyslipidemia risk in patients with TC compared with the controls was 0.822 (95% CI, 0.534-1.266), consistently showing no significant difference between the two groups.

**Table 4:** Risk of dyslipidemia compared between the patients with thyroid cancer and controls.

Model	Sample size, N		HR or OR (95% CI)	P-value
	TC	Control		
**Prospective concept**				
Unadjusted model	193	466542	1.102 (0.878-1.383)	0.4027
Multivariable-adjusted model	114	212419	0.932 (0.707-1.230)	0.6212
Propensity score-adjusted model				
Stratification	114	212419	0.964 (0.731-1.273)	0.7982
Within propensity score tertile				
1 (Lowest propensity score)	6	70838	1.473 (0.554-3.917)	0.4381
2	30	70815	0.96 (0.588-1.567)	0.8702
3 (Highest propensity score)	78	70766	0.927 (0.648-1.325)	0.6766
Regression adjustment	114	212419	0.938 (0.711-1.238)	0.6501
Weighting (stabilized IPTW)	114	212419	1.012 (0.838-1.223)	0.8976
Matching	114	570	0.818 (0.598-1.12)	0.2101
**Retrospective concept**				
Nested case-control study	170	277864	0.822 (0.534-1.266)	0.3734

HR, hazard ratio; OR, odds ratio; CI, confidence interval; IPTW, inverse probability treatment weighting.

## Discussion

In this large population-based cohort study, no significant difference in dyslipidemia risk was observed between patients with TC and the general population. This finding was consistently observed in both the prospective and retrospective analyses. First, the prospective analysis assessed dyslipidemia risk after TC diagnosis compared to patients without TC, then the retrospective analysis complemented the initial outcome by checking whether there was a difference in TC risk between patients with dyslipidemia and those without.

Thyroid function significantly affects lipid metabolism, including synthesis, mobilization, and degradation (12). Hypothyroidism is associated with dyslipidemia, and the restoration of normal TSH levels with l-thyroxine is beneficial for patients with overt hypothyroidism, as it improves their lipid profiles (13, 26). A meta-analysis showed that subclinical hypothyroidism is two to three times more frequent in patients with hypercholesterolemia, and that cholesterol levels are slightly elevated in patients with subclinical hypothyroidism (27). In contrast, hyperthyroidism can be associated with acquired hypocholesterolemia or an unexplained improvement in the lipid profiles of patients with dyslipidemia (28, 29).

However, it remains controversial whether patients with TC have an increased risk of dyslipidemia after cancer treatment. In an Israeli cohort study, TC survivors showed higher all-cause mortality with a higher prevalence of dyslipidemia and CVD than matched individuals without TC (17). Similarly, Li et al. showed that after thyroidectomy, the risk of dyslipidemia markedly increased in patients with differentiated TC (22). When the changes in serum cholesterol levels were evaluated according to postoperative TSH levels, patients receiving levothyroxine after total thyroidectomy with normal TSH levels had a higher risk of hypercholesterolemia than did those with mildly suppressed TSH levels (18). In contrast, a recent systematic review and meta-analysis showed that dyslipidemia, including both hypercholesterolemia and hypertriglyceridemia, was not associated with TC (16). Further, Chu et al. found no differences in the levels of cholesterol, LDL-C, triglyceride, and HDL-C before and after surgery in patients with TC, regardless of treatment modality (23).

Nevertheless, most previous studies were conducted on a relatively small number of patients over a short period of time, whereas our study was based on a large cohort database with a follow-up period of over 7 years. In this study, we found no relationship between the risk of dyslipidemia and TC. The exact mechanism behind this finding remains unclear, but many factors should be considered, such as the type of surgery, dose of radioactive iodine treatment, or the general practice of postoperatively suppressing TSH levels inducing subclinical hyperthyroidism to variable grades (30). The therapeutic response to levothyroxine may also differ according to an individual’s physical status. Interestingly, in line with our study, a recent study also specifically evaluated the effects of restoration of euthyroidism after long-term exogenous subclinical hyperthyroidism in patients with differentiated TC and found it had no significant influence on lipid and glucose parameters (24).

Another factor to consider is the phenomenon of over-diagnosing early, very small (< 1cm) TC cases in recent years. TC diagnosis often happens incidentally after the performance of radiologic tests for other conditions (31), and this phenomenon is particularly relevant in Korea (32) secondary to an extensive screening procedure. This phenomenon could reduce the differences between TC patients and the general population.

To conclusively answer the question whether TC is associated with dyslipidemia, much larger and longer-term studies are needed. These studies should consider the effects of various treatment modalities, both surgical and non-surgical such as levothyroxine dosage or other medications, TSH levels, as well as the duration of subclinical hyperthyroidism, and should investigate whether the restoration of euthyroidism influences lipid metabolism over both short and long terms. Further, various studies have postulated various associations between TC and cardio-cerebrovascular diseases; it was recently suggested that TSH suppressive therapy could increase cardio-cerebrovascular risk especially in high risk subjects (30, 33, 34). In light of our findings, the increased risk may be independent from dyslipidemia, a classical risk factor for cardio-cerebrovascular disease and warrants additional vigorous research.

### Strengths and Limitations

Our study has a few limitations. Due to the lack of thyroid function test values, we did not evaluate the potential difference between patients with euthyroidism and subclinical hyperthyroidism, which could affect lipid values. We also did not include clinicopathological characteristics such as the subtypes or stages of TC, but the majority of patients with TC in Korea (up to 97%) are classified as having differentiated TC (especially papillary TC) (1, 35). Lastly, additional studies are warranted to increase statistical power with a larger number of cancer patients.

The major strengths of this study are that we used a large real-world database representative of the Korean population, with an effective wash-out period of 7 years (2002–2008) and an extended follow-up period of 7 years (2009-2015), which have not yet been possible in the few previous studies on this topic. Moreover, we presented combined information from the bidirectional analysis in order to improve the inferences on the relative hazard of dyslipidemia events. Although the advantages of prospective cohort studies include the possibility of examining multiple results from a given exposure, determining disease rates in exposed and unexposed individuals over time, the main disadvantage of such prospective studies is that they require a large number of individuals to be followed up for long periods of time (36) and may result in biases, especially if significant loss occurs during follow-up (37). The nested case-control study reduces selection bias, as it matches cases with controls who have not developed the disease at the time of disease occurrence in the cases, thereby allowing the length of follow-up to be matched in addition to other variables. This allows for complementation of the general prospective analysis study (38, 39). Further, this relationship has not yet been explored in East-Asian populations.

## Conclusion

This large population-based cohort study with a 7-year follow-up period revealed no significant difference in dyslipidemia risk between patients with TC and the general population. To the best of our knowledge, this is the first study to perform bidirectional analyses of the risk of dyslipidemia in patients with TC, as well as the first East-Asian study overall to perform such an assessment. Nevertheless, further studies are warranted to explore the effect of cancer treatment on lipid metabolism and the difference between short- and long-term substitution therapy. In addition, future studies should aim to identify the exact relationship between TC and dyslipidemia and clarify the underlying mechanisms.

## Data Availability Statement

Restrictions apply to the availability of some or all data generated or analyzed during this study to preserve patient confidentiality or because they were used under license. The corresponding author will on request detail the restrictions and any conditions under which access to some data may be provided.

## Ethics Statement

The studies involving human participants were reviewed and approved by Institutional Review Board of Yonsei University Gangnam Severance Hospital. Written informed consent for participation was not required for this study in accordance with the national legislation and the institutional requirements.

## Author Contributions

JL, S-WK, YS, and HL contributed to conception and design of the study. JL and S-WK acquired the data, GP organized the database, and HL and GP performed statistical analysis. JL, S-WK, and YS interpreted the data. YS and HS drafted the manuscript; JL, S-WK, and GP provided critical revision. All authors have revised and approved the submitted manuscript.

## Funding

This study was supported by the Technology Innovation Program [20002781, A Platform for Prediction and Management of Health Risk Based on Personal Big Data and Lifelogging] funded by the Ministry of Trade, Industry & Energy (MOTIE, Korea) and was supported by the Korea Institute of Planning and Evaluation for Technology in Food, Agriculture and Forestry (IPET) through High Value-added Food Technology Development Program funded by Ministry of Agriculture, Food and Rural Affairs (MAFRA) (321030051HD030).

## Conflict of Interest

The authors declare that the research was conducted in the absence of any commercial or financial relationships that could be construed as a potential conflict of interest.

## Publisher’s Note

All claims expressed in this article are solely those of the authors and do not necessarily represent those of their affiliated organizations, or those of the publisher, the editors and the reviewers. Any product that may be evaluated in this article, or claim that may be made by its manufacturer, is not guaranteed or endorsed by the publisher.
